# Role of Inner Ear Macrophages and Autoimmune/Autoinflammatory Mechanisms in the Pathophysiology of Inner Ear Disease

**DOI:** 10.3389/fneur.2022.861992

**Published:** 2022-04-06

**Authors:** Toru Miwa, Takayuki Okano

**Affiliations:** ^1^Department of Otolaryngology-Head and Neck Surgery, Graduate School of Medicine, Kyoto University, Kyoto, Japan; ^2^Department of Otolaryngology-Head and Neck Surgery, Tazuke Kofukai Medical Research Institute Kitano Hospital, Osaka, Japan

**Keywords:** macrophages, monocytes, heterogeneity, autoimmune inner ear diseases, autoinflammatory diseases, colony-stimulating factor 1

## Abstract

Macrophages play important roles in tissue homeostasis and inflammation. Recent studies have revealed that macrophages are dispersed in the inner ear and may play essential roles in eliciting an immune response. Autoinflammatory diseases comprise a family of immune-mediated diseases, some of which involve sensorineural hearing loss, indicating that similar mechanisms may underlie the pathogenesis of immune-mediated hearing loss. Autoimmune inner ear disease (AIED) is an idiopathic disorder characterized by unexpected hearing loss. Tissue macrophages in the inner ear represent a potential target for modulation of the local immune response in patients with AIED/autoinflammatory diseases. In this review, we describe the relationship between cochlear macrophages and the pathophysiology of AIED/autoinflammatory disease.

## Introduction

The functional mechanisms of resident macrophages (Mφs) in the inner ear remain largely unknown, in both the context of normal tissue physiology and disease development. Several recent reviews have discussed the importance of tissue-resident Mφs in the systemic and local regulation of inner ear homeostasis and disease pathology that improve our understanding of inner ear-resident Mφs (1–6).

In the United States, over 30 million adults, or approximately 15% of the entire population, are affected by hearing loss (7, 8). Research has indicated an expansion of the populations affected by sensorineural hearing loss, which impacts the conversion of mechanical sound to the neuroelectric indicators in the inner ear that relay signals to the auditory nerve (7, 8). Sensorineural hearing loss exerts a life-changing impact on affected patients; moreover, the current treatment modalities for sensorineural hearing loss are limited to the use of either cochlear implants or hearing aids. Cochlear implants directly stimulate the cochlear nerve by bypassing the damaged organ of Corti, which is most frequently affected by sensorineural hearing loss (9). Cochlear implants are among the artificial organs with the highest success rates; however, there is a social demand for more fundamental therapeutic options for sensorineural hearing loss.

Autoimmune inner ear disease (AIED) represents much fewer than 1% of all cases of sudden sensorineural hearing loss (10, 11), generally presenting as subacute, swiftly progressing, frequently fluctuating, bilateral, and asymmetrical sensorineural hearing loss (9–14). AIED can be labeled as primary AIED, in which the inner ear is the most affected organ, or secondary AIED (15–30% of cases), which develops in association with a systemic autoimmune disease (12), such as autoimmune hepatitis (15), systemic lupus erythematosus (SLE) (16), multiple sclerosis (MS) (17), rheumatoid arthritis (18), inflammatory bowel disease (IBD) (19), or antiphospholipid syndrome (14, 20).

Collectively, current evidence indicates that aberrant events in the early innate immune response play critical roles in the development and manifestation of autoimmune hearing loss (10, 11). As with most autoimmune diseases, it has been postulated that a misdirected assault on the self-organs, mainly inner ear proteins in AIED, activates the pro-inflammatory T-cell response and autoantibody formation; this represents the basic pathophysiology of AIED and other autoimmune diseases. Autoinflammatory diseases fall within the family of immune-mediated diseases, many of which induce sensorineural hearing loss, suggesting that similar mechanisms are involved in the pathogenesis of AIED (21). In the autoinflammatory disorder-related hearing loss, genetic mutations or polymorphisms inherited in an autosomal dominant manner result in a gain-of-function mutation within the gene for nod-like receptor (NLR) family pyrin domain 3 (NLRP3), leading to excessive interleukin (IL)-1β release, sensorineural hearing loss, systemic amyloidosis, and/or transient skin rashes. Muckle–Wells syndrome (MWS) and neonatal-onset multisystem inflammatory sickness belong to a family of autoinflammatory diseases known as a cryopyrin-related periodic syndrome, which also involves sensorineural hearing loss (22, 23). However, the role of Mφs in AIED and autoinflammatory diseases has not been well-documented thus far.

Although 70% of patients with AIED initially respond to corticosteroids (11), understanding the role of Mφs in the pathophysiology of progressive hearing loss is critical for the development of improved therapies. In this review, we discuss recent findings related to the development of immune-competent cells within the inner ear (24, 25), thereby clarifying the role of inner ear-resident Mφs in inner ear homeostasis and pathological processes. We also cover a wide range of research areas involving tissue-resident Mφs that include those related to recent advances in antigen differentiation, gene expression patterns, and the clinical features and pathology of AIED/autoinflammatory diseases.

## Origins and the Diversification of Tissue-Resident Macrophages—Identification Through Differentiation of Antigens, Fate-Mapping, and Gene Expression Patterns

**Figure 1:**
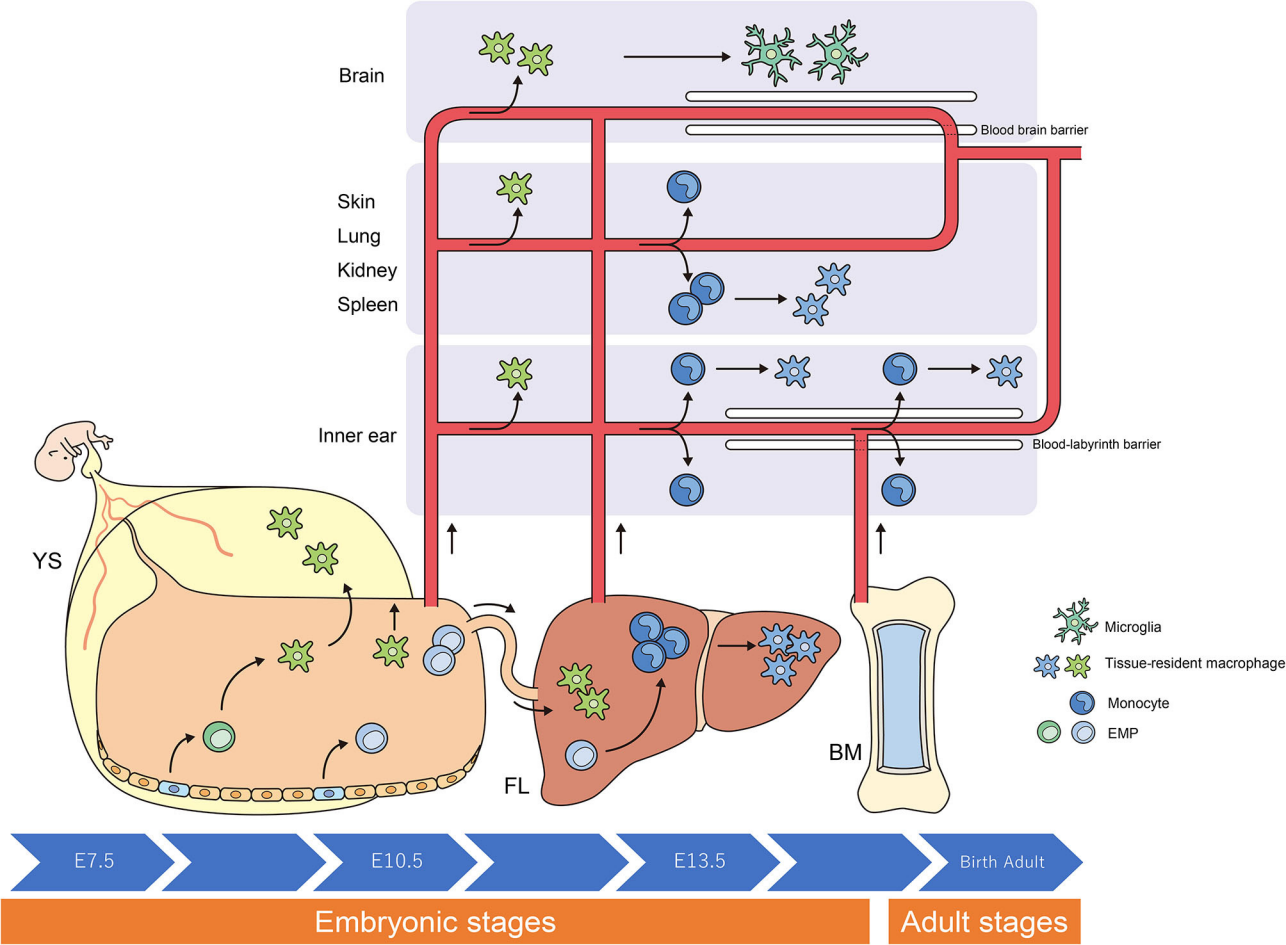
Origins and renewal of tissue-resident macrophages. The latest destiny-mapping studies in mice imply that Mφs derived from the YS during early embryogenesis contribute to pools of mature tissue Mφs that include Langerhans cells and microglia. Further seeding in different tissues occurs following definitive hematopoiesis in the FL or BM. Proliferative local expansion of tissue Mφs within the neonatal period, followed by low-level self-renewal throughout adulthood, appears sufficient for maintaining many tissue-resident Mφ populations. By helping to resolve acute inflammation, local proliferation may contribute to restoring homeostatic tissue-resident Mφ populations. Although the exact contribution of BM-derived inflammatory Mφs to these tissue-resident pools remains unclear, it nonetheless appears to take place, perhaps in a tissue-specific manner. YS, yolk sac; FL, fetal liver; BM, bone marrow; EMP, erythro-myeloid progenitor; Mφ, macrophage.

Macrophages are present in all vertebrate tissues, emerging earlier than any other blood cell type from mid-gestation and are distributed in almost every organ and tissue in the body throughout life (26, 27). In addition to their role in regulating tissue development and regeneration, Mφs aid in maintaining local homeostasis by responding to internal and external stimuli, appearing as phagocytes that protect against microbes. Furthermore, they participate in the clearance of useless and senescent cells and act as sentinels with trophic, regulatory, and repair functions. Heterogeneous Mφ phenotypes are observed in different tissue environments that highlight their organ-specific capabilities in developmental processes and normal physiology (28–30) (Figure 1). In addition, Mφs exhibit diverse tissue-specific functions, integrating cues from the external surroundings and their microenvironment. Hence, tissue-resident Mφs represent an appealing target for therapeutics given their implication in various pathological processes that include those related to atherosclerosis, autoimmune diseases, neurodegenerative and metabolic disorders, and tumor growth (13). Elucidating the developmental pathways and characteristics of Mφs may aid in the design of novel interventional strategies, which focused on the tissue-specific microenvironment.

Researchers have debated whether resident Mφs are constantly and predominantly repopulated *via* the delivery of blood-circulating monocytes, which derive from progenitors inside the bone marrow (BM). However, recent studies have demonstrated that specific Mφ populations are independent of circulating monocytes and even adult BM hematopoiesis (1, 2, 24, 31). These tissue-resident Mφs are derived from the sequential seeding of tissues by means of various precursors during embryonic development. Primitive Mφs are generated from early erythro-myeloid progenitors (EMPs) inside the yolk sac (YS) that bypass monocytic intermediates and give rise to microglia *via* the transcription element *c-Myb*. Ultimately, fetal monocytes are generated from *c-Myb*+ EMPs that begin to seed the fetal liver (FL), giving rise to various types of mature Mφs. Hence, hematopoietic stem cell-impartial embryonic precursors transiently are present in the YS and FL represents the origin of long-lasting, self-renewing Mφ populations with organ-specific functions (1, 26) (Figure 1).

The percentage of resident Mφs varies according to their origin, the developmental stage of the organism, and the tissue type. For instance, most of the microglia in the brain originate from YS-derived Mφs, whereas Mφs from the FL and BM provide a negligible contribution to microglia during all stages of life (6, 10). In contrast, resident Mφs in the gut are derived from the YS at some point during early embryonic development. Monocytes derived from the FL give rise to most of the resident Mφs in the intestine at birth; however, throughout adulthood, most resident Mφs originate from the BM (5, 27).

**Figure 2:**
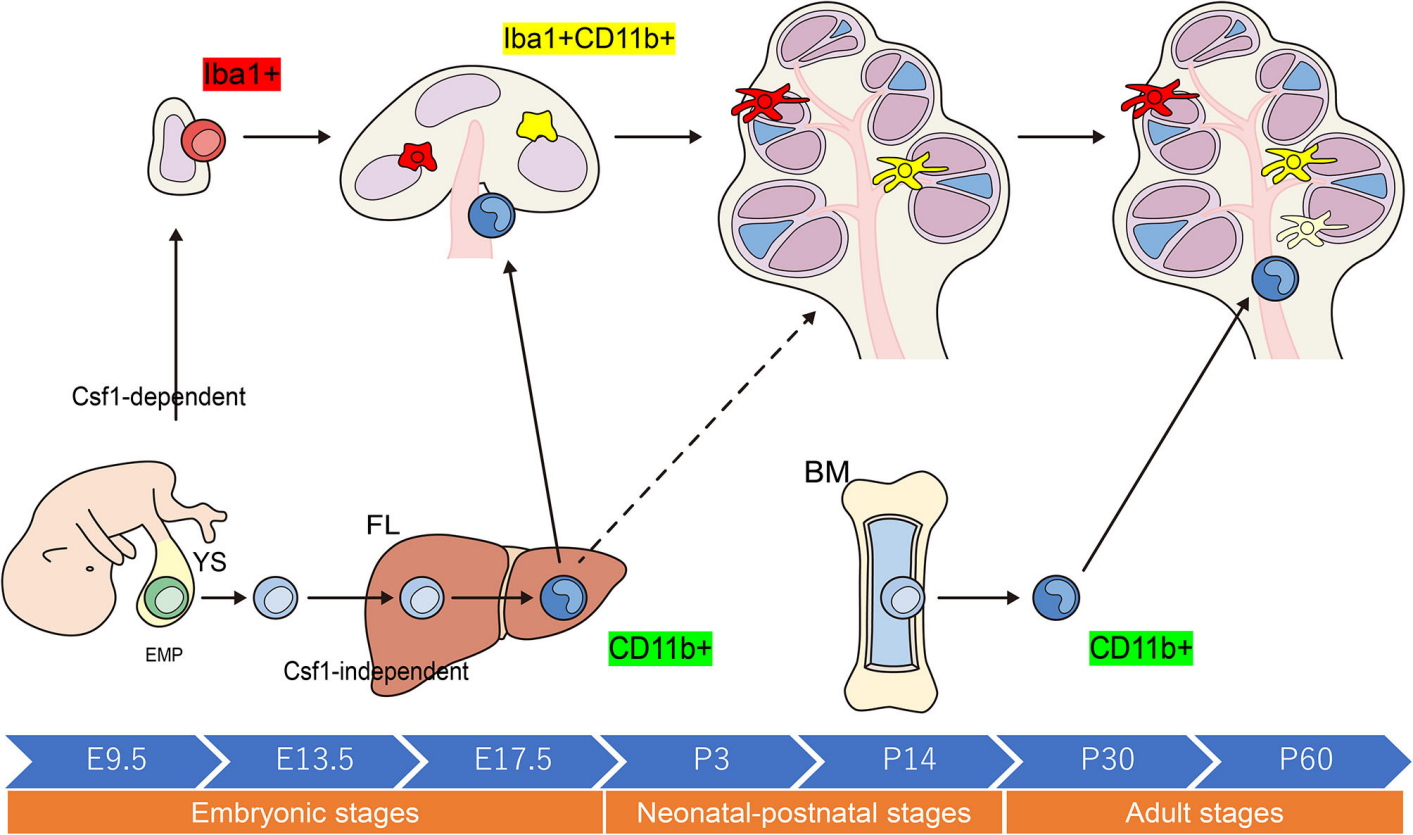
Schematic representation of the origins and distribution of resident macrophages in embryonic and adult cochleae. Two subtypes of resident Mφs are present within the embryonic cochlea: Csf1r-dependent Mφs that originate from the YS and Csf1r-independent Mφs that migrate from the FL via systemic circulation. A large proportion of the cochlear-resident Mφ population is derived from the YS given that Mφs expressing Iba1 reside in the mesenchyme surrounding the otocyst as early as E10.5. These Mφs are distributed within the SGN and SLi at E17.5. However, Csf1r-independent Mφs expressing CD11b migrate as early as E14.5 and reside only in specific components of the cochlea, consisting of the mesenchyme of the cochlear modiolus or the intraluminal floor of the perilymphatic area at E17.5. In the adult cochlea, the density of Mφs expressing Iba1 regularly decreases, whereas that of Mφs expressing CD11b increases, suggesting that the FL and BM contribute to the repopulation of cochlear-resident Mφs. YS, yolk sac; FL, fetal liver; BM, bone marrow; Mφ, macrophage; Csf1r, Colony-stimulating factor-1 receptor; SGN, spiral ganglia neurons; SLi, spiral ligament; EMP, erythro-myeloid progenitor.

**Figure 3:**
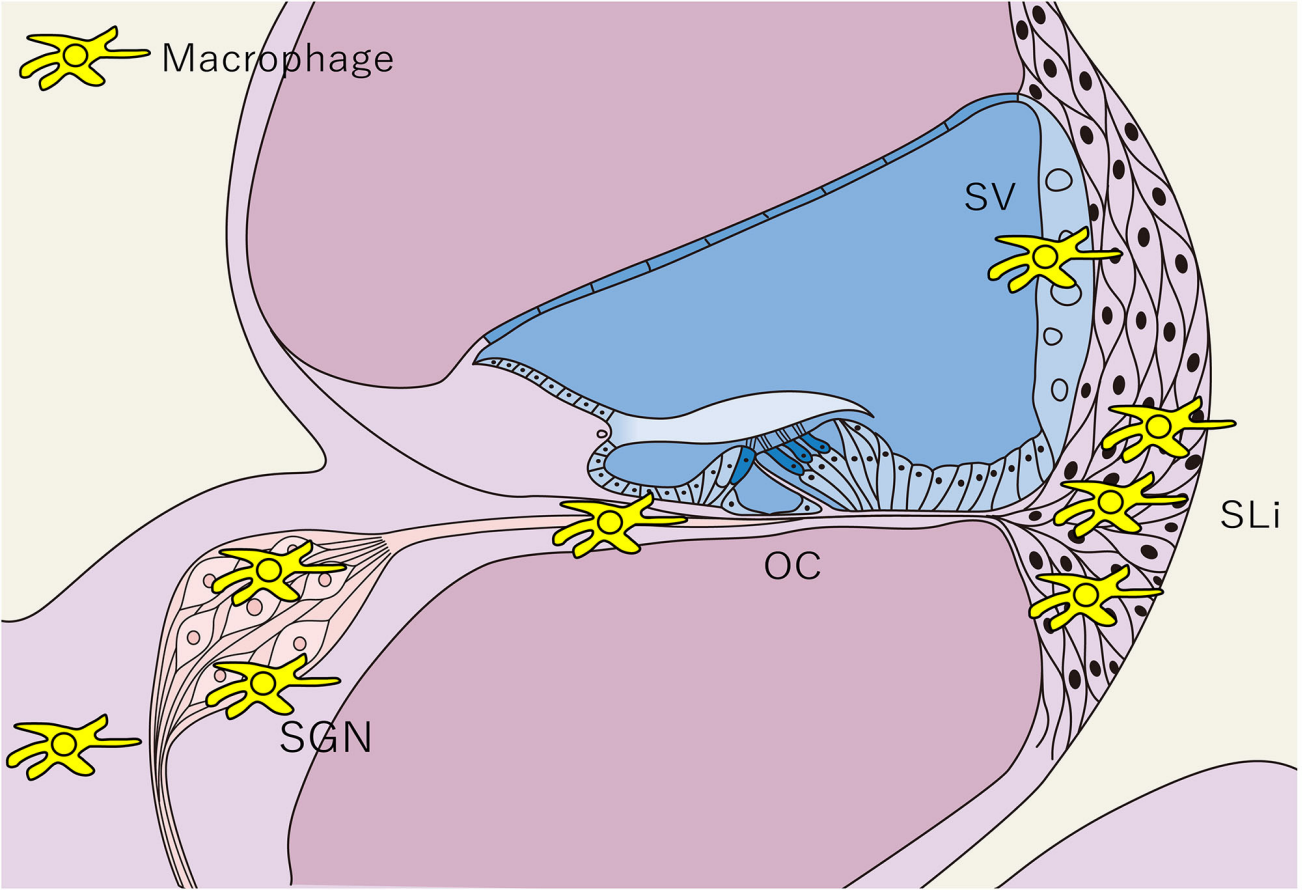
Distribution of tissue macrophages in the cochlea. The distribution of tissue Mφs is shown in a schematic cross-sectional view of the cochlea. Tissue Mφ are distributed in the SGN, SLi, SV, and cochlear modiolus. Cochlear Mφs also exhibit spindle-shaped cell bodies and dendritic cell processes under steady-state conditions, as observed in microglia in the central nervous system. SGN, spiral ganglion neurons; SLi, spiral ligament; SV, stria vascularis; OC, Organ of Corti; Mφ, macrophage.

When compared with other tissue-resident populations of Mφs, the cellular expression profiles of cochlear Mφs and the markers that can be used to visualize these cells have been poorly documented. The dynamics of Mφ populations in the developing cochlea have been characterized most appreciably in mice and are summarized in Figure 2 (24, 31, 32). Colony-stimulating factor 1 (Csf1) signaling controls the seeding of the larger Mφ population within the cochlea throughout development (24, 25). A second populace derived from the FL, which is Csf1 receptor-independent, is observed in the modiolus and the intraluminal surface of the perilymphatic area inside the embryonic cochlea (24). Cochlear Mφs and perivascular macrophage-like melanocytes (PVM/Ms) lie close to blood vessels in the adult cochlea (33)—such as in the cochlear modiolus, supporting cells, spiral ganglion neurons (SGNs), stria vascularis (SV), and spiral ligament (SLi)—under normal conditions (4, 24, 25, 31, 32, 34–36) (Figure 3).

The inner ear is responsible for auditory sensation and the perception of acceleration/rotation, and it is difficult to restore the population of sensory cells in the inner ear after degeneration due to acute or chronic inner ear injuries, such as those related to Meniere's disease and sudden sensorineural hearing loss. This is partly because, in humans, hair cells in the inner ear are terminally differentiated, losing their potential for self-renewal following significant damage after birth (33). SGNs, which mediate synaptic connections among the hair cells and the neurons of the cochlear nucleus, also undergo damage and degeneration, while damage or atrophy of the SV and SLi disrupts cochlear function. Repeated exposure of the auditory system to insults causes harm to these structures, resulting in functional impairments that lead to progressive hearing loss (7).

Cochlear Mφs persist from the early post-natal stages and renew or preserve their populace *via* the infiltration of circulating monocytes (34, 37, 38). PVM/Ms are found adjacent to the blood vessels in the SV and support cells in the cochlea (35, 39). PVM/Ms have a turnover time of several months in mice and are maintained *via* the migration of monocytes that originate from the BM into the cochlea (36). Functionally, PVM/Ms contribute to restoring the endocochlear potential, which is crucial for the activation of auditory hair cells (40, 41). Interestingly, Mφ heterogeneity results in distinct phenotypes and, more importantly, completely exceptional organic functions in other tissues (28). Therefore, future studies should aim to elucidate the roles of tissue-resident and BM-derived Mφs in the initiation, progression, and termination of inner ear diseases.

## Macrophages and Autoimmune Diseases

Monocytes and Mφs can secrete a wealth of cytokines and chemokines, which further stimulate other forms of immune cells, thereby leading to inflammation (33, 42). The presence of autoantibodies and autoreactive B and T cells in most autoimmune diseases indicates that the adaptive immune system is essential for their pathogenesis; however, this cannot completely account for the resolution and development of those diseases, and studies have indicated that the innate immune response may also play necessary and irreplaceable roles in the pathogenesis of the autoimmune disease (33, 43, 44).

A monocyte or Mφ infiltration is typically observed in most autoimmune diseases (33). The regulatory mechanisms that involve monocytes and/or Mφs in the development of the autoimmune disease have not been fully elucidated; nevertheless, the consensus appears to signify that their atypical activation plays a key role in the abovementioned mechanisms. Mφs exhibit shifts in polarization primarily based on diverse stimuli produced with the aid of cytokines, microbes, microbial products, and other modulators *in vitro* (45). However, in an *in vivo* study, alterations in arginine metabolism following lipopolysaccharide (LPS) injection were found to elicit different phenotypes of Mφs in C57BL/6J and Balb/c mice (46). C57BL/6J peritoneal Mφs promoted inducible nitric oxide synthase (iNOS) activity, resulting in nitric oxide expression and a T-helper 1 (Th1) CD4+ T cell response. In contrast, Mφs from Balb/c mice triggered arginase activity, resulting in an ornithine and Th2 response. Analogous to Th1 and Th2, these Mφs have been termed M1 and M2 (46). Generally, M1-polarized Mφs are pro-inflammatory and secrete IL-12 and tumor necrosis factor (TNF)-α, which contribute to local irritation, while M2-polarized Mφs produce IL-4 and IL-10, which play roles in immunomodulation, wound repair, and tissue remodeling (43, 47).

Within tissues of the central nervous system (CNS), particularly in patients with progressive MS, infection is characterized by the massive activation of mononuclear phagocytes that include both monocyte-derived Mφs and resident microglia (48). The staging of MS lesions can be determined based totally on the presence of CD68-nice Mφs and human leukocyte antigens, together with the extent of myelin loss (49). Findings obtained using the experimental autoimmune encephalomyelitis (EAE) model, an animal model of MS, have indicated that Mφs play crucial roles in triggering adaptive immune responses. For example, the Mφ NLRP3 inflammasome plays key role in inducing autoreactive T cell migration into the CNS in EAE (50). Mφs also produce several key cytokines (IL-1β, IL-6, and IL-23) that promote the generation and maintenance of Th17 cells, an important cell subset mediating CNS autoimmunity in EAE (51). For this reason, accumulating evidence suggests that Mφs play divergent roles in the pathogenesis of MS, exacerbating tissue damage despite their outstanding growth-promoting and neuroprotective effects (52). As predicted, this dual role of Mφs in MS may be defined by their polarization state. Indeed, both M1 and M2 subsets are found in MS lesions. The unexpected pro-inflammatory M1 reaction is maintained at sites of CNS damage, while the immunoregulatory M2 response is comparatively weaker and more transient (52). Yamasaki et al. demonstrated that resident macroglia are associated with particle clearance. In the context of MS, these cells exhibit global suppression of metabolism throughout disease initiation, whereas monocyte-derived Mφs become exceptionally phagocytic and inflammatory, actively participating in the initiation of demyelination (53).

Monocytes/Mφs contribute to the pathogenesis of SLE by modulating the adaptive immune response in the kidney. Defective Mφ phagocytosis has also been thought to contribute to autoimmunity in SLE. The phagocytic potential of Mφs is vital for the clearance of dead cells and debris, which otherwise may be critical sources of autoantigens. Accumulating evidence from *in vitro* studies and murine models illustrates that ineffective clearance of apoptotic cells by Mφs may represent a critical trigger of the autoimmune response in SLE (33). In lupus-inclined NZB/W and NZW/BXSB mice, resident nephritic Mφs exhibit decreases in arginase and iNOS production despite treatment with M1- or M2 Mφ-inducing cytokines, regardless of their health status (54). Instead, these Mφs exhibit a combined pro- and anti-inflammatory phenotype throughout the course of lupus-related nephritis. The authors argued that monocyte-derived Mφs in these mice are poorly responsive to the cytokine stimulation that enables the transition to the corresponding M1 or M2 type (54). In addition to these phenotypic differences, functional analysis has confirmed that resident kidney Mφs exhibit greater antigen-producing and phagocytotic effects than monocyte-derived kidney Mφs (33, 54).

Autoimmune uveitis, which occurs in several diseases that include Behçet's disease, sarcoidosis, and Vogt–Koyanagi–Harada disease, is a sight-threatening ocular inflammatory disorder (55). Immunization with interphotoreceptor retinoid-binding protein and extra adjuvants leads to the priming of autoreactive CD4+ T cells in peripheral lymphoid organs and their polarization into pathogenic Th1 and Th17 cells. Once activated, Th cells in the eye induce the breakdown of the blood–retinal barrier, an immune barrier that protects the eyes from unfavorable inflammation through tight junctions between endothelial cells in blood vessels; these tight junctions block circulating leukocyte extravasation into the retina (56). Okunuki et al. suggested that retinal microglia constitute the essential cellular populace within the retina that enables entry of the autoreactive cells required for the initiation of autoimmune uveitis; however, systemic exposure to an autoantigen is in all likelihood the cause of autoimmunity in this disorder (55).

Taken together, these findings highlight the proposed relationship between monocytes/Mφs and the development of autoimmune diseases in the CNS, kidneys, and eyes. It is well-known that monocytes/Mφs are the key components of the innate immune system that underlie the amplification and suppression of inflammation (42). Increasing evidence indicates that these cells participate in the pathogenesis of autoimmune diseases mainly *via* their remarkably pro-inflammatory or fibrogenic functions (42, 57). As discussed above, the heterogeneity of monocyte/Mφ subpopulations varies dramatically in different autoimmune diseases, and their polarization profiles generally play key roles in diseases progression. However, for several autoimmune diseases, the phenotypic and functional characteristics of monocytes/Mφs remain poorly categorized, as many pro-inflammatory M1-polarized monocytes/Mφs concurrently express M2-associated markers or showcase immunomodulatory features (33).

## The Role of Inner Ear Macrophages in AIED

The inner ear is fully able to mount an immune response following the invasion of outside antigens. Consequently, numerous mechanisms have been proposed to underlie cochlear damage that include antibody–antigen reactions with autoantibody enhancement (type II immune responses), complement machine activation, immune-complicated depletion (type III immune responses), direct damage mediated *via* cytotoxic T-cells crossing the blood–labyrinth barrier and reaching the endolymphatic sac, vasculitis, micro-thrombosis, and electrochemical reactions (type IV immune responses) (10, 12, 14, 20, 58). Antigen recognition by the innate immune cells of the inner ear (neutrophils, Mφs, and dendritic cells) stimulates the release of IL-1β, which in turn triggers a series of adaptive immune responses. The recruitment of immunocompetent cells and the promotion of an adaptive immune response occur in the presence of immune mediators, such as IL-1β, IL-2, and TNFα (13, 20). Studies have suggested an association between sudden sensorineural hearing loss and the presence of vestibulocochlear antibodies against inner ear antigens, such as heat shock protein 70, cochlin, β-tectorin, and types II and IX collagen (12, 20, 59–61).

Although the relationship between AIED/autoinflammatory diseases and cochlear monocytes/Mφs remains largely unknown, previous studies have indicated that cochlear Mφs play crucial roles in the onset and progression of infection after insults to the cochlear sensory epithelium. Such insults include noise or drug exposure and mechanical or surgical injury to the cochlea, such as that occurring during cochlear implantation (5, 31, 34, 62–72). These signals activate resident cochlear Mφs, switching them to a pro-inflammatory state and causing them to release cytokines that recruit monocytes into the cochlea (34, 62). Infiltrating monocytes then differentiate into macrophages within the cochlea, following which they exert phagocytotic functions (69, 73), produce inflammatory mediators, and play roles in antigen presentation (34). In some patients with Ménière's disease, high levels of IL-1β and TNF-α suggested that it was a chronic inflammatory disorder (74). In addition, several reports suggested an association between the immune responses caused by Mφs and Ménière's disease (75–80).

As described above, monocytes/Mφs are key components of the innate immune system in the pathogenesis of systemic autoimmune diseases and are involved in amplifying and suppressing inflammation (33, 42), mainly through their remarkably pro-inflammatory or fibrogenic properties (33, 42, 57). Nakanishi et al. suggested that monocytes are the primary regulators of IL-1 release in MWS, which is caused by a mutation in the *NLRP3* gene that leads to excessive IL-1β production (81). Gattorneo et al. also reported that monocytes from patients with MWS released a minute amount of IL-1 when compared with control monocytes, but that patients were extremely responsive to anakinra use (IL-1 inhibition) (82). Additionally, Nakanishi et al. indicated that LPS stimulation can activate the NLRP3 inflammasome in monocyte/Mφ-like cells (CX3CR1-superb cells) in wild-type C57BL/6J mouse cochleae (81). These findings support the notion that local cochlear activation of the NLRP3 inflammasome in monocytes/Mφs can result in cochlear autoinflammation and sensorineural hearing loss (83). Vambutas et al. demonstrated that patients with steroid-resistant AIED are clinically sensitive to IL-1 inhibition; however, relative to those in individuals with steroid-sensitive AIED and controls, these monocytes synthesize greater but release much less IL-1 (84).

Previous studies have revealed that 46–57% of adult patients with IBD present with sensorineural hearing loss as an extraintestinal manifestation (85, 86); however, few reports have mentioned the roles of monocytes/Mφs. Dettmer et al. reported that the temporal bones of patients with IBD exhibited mild chronic inflammation, poorly defined granulomas, and CD68-positive Mφ infiltration (87). IBD may also be associated with Cogan's syndrome, a rare disorder characterized by eye and inner ear inflammation manifesting as interstitial keratitis and audiovestibular dysfunction, respectively (88). The mechanisms associated with eye and inner ear disorders in Cogan's syndrome are unknown; however, the authors of one autopsy case reported histopathologic evidence of vasculitis and an infiltration of CD45-positive inflammatory cells that include Mφs in both the cochlear and peripheral vestibular systems (89). Moreover, a few studies have suggested that the disease is a result of inner ear autoimmunity (90, 91). Aberrantly activated intestinal Mφs in patients with IBD produce diverse cytokines (IL-1β, IL-6, IL-23, TNF-α, and TNF-like protein 1A) required for T-cell differentiation, especially those related to the generation of Th1 and Th17 cells (92). Furthermore, in those with IBD, intestinal Mφs cause an abnormally fast breakdown of pro-inflammatory cytokines due to faster lysosomal degradation, whereas cytokine mRNA expression remains stable and within the normal range (93). This has been shown to elicit an impaired neutrophil response, leading to dysfunction in bacterial clearance and thereby boosting the formation of granulomas. Within the pathology of IBD, Mφs are hyperpolarized toward the M2 profile, as demonstrated in numerous studies. In various mouse models of IBD, inhibition of the pro-inflammatory activities of M1 Mφs or induction of tissue-repairing/immunomodulatory M2 Mφs results in attenuated experimental IBD (94, 95). Similar mechanisms are speculated to occur in the inner ear in patients with IBD.

**Figure 4:**
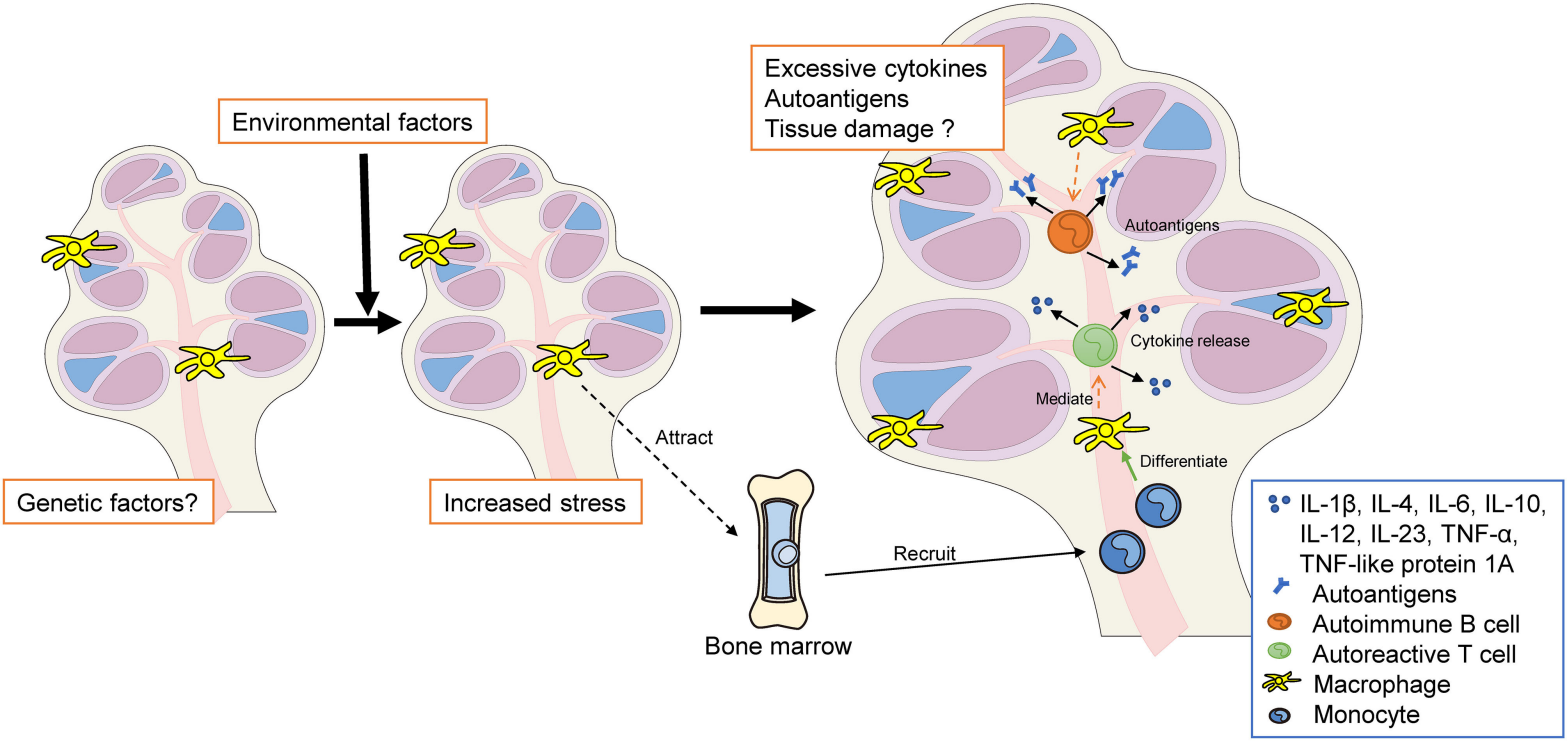
The hypothesis of autoimmune and autoinflammatory diseases in the inner ear. Genetic and/or environmental factors increase both systemic stress and local stress in the inner ear (e.g., MS, SLE, autoimmune uveitis, MWS, and IBD). Resident cochlear Mφs may be activated to a pro-inflammatory state. Pro-inflammatory macrophages release cytokines that recruit monocytes into the cochlea. These infiltrating monocytes differentiate into macrophages within the cochlea, following which they participate in phagocytosis, inflammatory mediator production, and antigen presentation. Excessive autoantigen and cytokine release may cause cochlear autoinflammation and sensorineural hearing loss. MS, multiple sclerosis; SLE, systemic lupus erythematosus; MWS, Muckle–Wells syndrome; IBD, inflammatory bowel disease; Mφ, macrophage, BM, bone marrow; TNF, tumor necrosis factor; IL, interleukin.

In summary, cumulative and progressive sensory cell degeneration and death, caused by chronic inflammation, end in the activation of resident Mφs, with little infiltration of circulating monocytes (62), which parallels the innate immune response observed in chronic diseases. The activated resident Mφs adopt either a pro- or anti-inflammatory profile. Pro-inflammatory Mφs produce and release pro-inflammatory mediators—such as IL-1β, TNF-α, and IL-6—which send signals to nearby cells, leading to further inflammation and cellular damage/apoptosis (34, 96, 97) (Figure 4). However, to date, no study has clarified which cells in the inner ear are targeted by Mφs, and the precise differences in phenotype and activity among newly recruited monocytes/Mφs and resident Mφs remain to be determined. Across autoimmune disorders, the heterogeneity of monocyte/Mφ subpopulations varies dramatically; furthermore, their polarization profile usually plays a key role in disease development (33). Further research is required to elucidate the specific roles of cochlear monocytes/Mφs in the pathophysiology of autoimmune-mediated hearing loss.

## Future Directions

Numerous research groups have proposed Mφ-targeted treatment options for inflammatory diseases. Among the most investigated strategies is the facilitation of Mφ phagocytosis *via* nanoparticle targeting, which then passively targets the inflammatory site due to the mounting immune response (98, 99). The modulation and reprogramming of Mφs are also considered promising anti-tumor strategies (69–71). Using genetically modified monocytes or Mφs as vectors may aid in the development of therapeutic strategies that promote regeneration or regrowth of particular structures inside the inner ear (6, 61, 100). This idea is especially well-suited for secreted paracrine or endocrine factors, such as hormones or growth factors. Because the inner ear includes three fluid-filled compartments, secreted elements derived from genetically modified Mφs may reach the central cells *via* diffusion through the inner ear without the involvement of the blood or lymphatic circulation. The transplantation of genetically engineered cells that can secrete particular metabolic or humoral cues may help to augment pharmacologic immune modulation inside the inner ear; however, the delivery of genetically modified cells into the inner ear may be challenging due to its anatomical characteristics. Given that monocytes and Mφs can migrate into the inner ear in both pathological and physiological states (6, 32, 61, 69), the human monocyte lineage can be extracted, cultured *ex vivo*, genetically manipulated, and reimplanted locally or systemically. Intravenous management of genetically modified monocytes should permit them to reach the inner ear despite challenges related to tissue or organ specificity.

Extending studies to identify and establish the set of markers expressed by cochlear Mφs to include multiplex and comparative transcriptomic studies may improve our ability to identify sub-populations and roles and to compare the profiles of resident Mφs in different organs. To better understand the roles of these cells, numerous processes that permit source- and area- (cochlear sub-structure) specific Mφ analysis are required. Such techniques can also be used to identify remote cells or tissue indicators *in situ*. For example, in the brain, infiltrating monocytes have been shown to exhibit functional variations and contribute to disease pathology *via* multiple mechanisms following ischemic stroke and MS (53, 101). To develop therapeutic strategies for AIED and autoinflammation, it is first necessary to understand the effects of targeting cochlear Mφs. Furthermore, as hearing loss fluctuates in patients with AIED and autoinflammation, the timing of intravenous administration of genetically modified monocytes may represent a potential problem in clinical applications.

## Conclusion

In this review, we discussed the latest advances and evidence regarding the connection between tissue-resident Mφs and AIED/autoinflammatory diseases. Three subtypes of cochlear-resident Mφs are found in exclusive components of the cochlea, and each subtype may play distinct roles in the abovementioned diseases. Furthermore, the dynamics and molecules expressed in tissue Mφs in the inner ear are gradually being elucidated. However, several aspects remain unclear. In particular, the role of tissue Mφs in the pathology of AIED is not well-understood, in part due to restrictions on the collection of human inner ear tissue. Moreover, additional research is required to compare the pathophysiology of such diseases in mouse models and humans. Despite the need for extensive research, the future of inner ear immunology and treatment for sensorineural hearing loss remains promising. Developing a greater understanding of monocyte/Mφ-associated activities within the cochlea may be essential for developing new biological and therapeutic strategies for related diseases.

## Author Contributions

TM designed and conducted the study and reported the findings. TO validated the study design, reviewed the manuscript, and supervised the study. Both authors contributed to the article and approved the submitted version.

## Funding

This work was supported by a JSPS KAKENHI grant (no. 19K09908) to TO. The funder had no role in the study design, data collection and analysis, publication decision, or manuscript preparation.

## Conflict of Interest

The authors declare that the research was conducted in the absence of any commercial or financial relationships that could be construed as a potential conflict of interest.

## Publisher's Note

All claims expressed in this article are solely those of the authors and do not necessarily represent those of their affiliated organizations, or those of the publisher, the editors and the reviewers. Any product that may be evaluated in this article, or claim that may be made by its manufacturer, is not guaranteed or endorsed by the publisher.
